# Effect of vitamins B_1_, B_6,_ and B_12_ (Neurobion) on Diisopropylfluorophosphate–induced Delayed Neuropathy in Mice

**Published:** 2018

**Authors:** Mohsen Ebrahimi, Mohammad Javad Khoushnoud, Majid Zia-Behbahani

**Affiliations:** a *Faculty of Medicine, AJA University of Medical Sciences, Tehran, Iran. *; b *Department of Pharmacology and Toxicology, School of Pharmacy, Shiraz University of Medical Sciences, Shiraz, Iran. *; c *Student Research Committee, Shiraz University of Medical Sciences, Shiraz, Iran.*

**Keywords:** Organophosphorus compound, DFP, Delayed neuropathy, Neuropathy target esterase, Neurobion

## Abstract

Certain organophosphorus esters, such as diisopropylfluorophosphate (DFP), cause delayed neuropathy by inhibition of neuropathy target esterase (NTE) keeping the neuron in normal function. In this study, effects of neurobion alone and in combination with dexamethasone on DFP–induced delayed neuropathy were evaluated. Thirty-five mice were divided into five groups, each consisting of 7 mice. Except group1 (Normal group), group 2 received normal saline and 1h later, 1 mg/kg DFP; groups 3, 4 and 5 received 150 mg/kg neurobion, 2 mg/kg dexamethasone and 150 mg/kg neurobion plus 2 mg/kg dexamethasone, respectively and 1h later 1mg/kg DFP. Twenty one days after the last injection, the mice were killed by decapitation under deep anesthesia. NTE level was determined in the brain and though there was no significant difference between the groups, neurobion and neurobion plus dexamethasone partly- not significantly (*p* > 0.05)- were able to prevent reduction of NTE in the brain caused by DFP. Histopathological evaluation of sciatic nerves showed that neurobion and neurobion plus dexamethasone significantly suppressed the harmful effect of DFP.

We also evaluated the activity of acetylcholine esterase (AChE), concentration of glutathione (GSH), and malondialdehyde (MDA) levels in the serum. Results showed dexamethasone

(*p* < 0.001) and dexamethasone in combination with neurobion (*p *< 0.01) diminished AChE activity significantly compared to the DFP group. Neurobion caused a significant increase in the GSH level (*p* < 0.05). No significant change was seen in MDA. It is suggested that neurobion should be added and used in the first aid equipment and techniques for exposure to organophosphorus compounds, e.g. pesticides and chemical warfare.

## Introduction

Organophosphorus (OP) esters are an important class of insecticides and chemical warfare agents (1). Their acute lethal action is attributed to inhibition of acetylcholinesterase by phosphorylation of its catalytic site (2). Organophosphorus compounds (OPs), such as tri-o-cresyl phosphate (TOCP), leptophos, diisopropylfluorophosphate (DFP) and triphenylphosphite induce delayed neuropathy [OP-induced DN, (OPIDN)] in addition to inhibition of acetyl choline esterase in human and other sensitive species (3-5).

OPIDN is caused by inhibition of neuropathy target esterase (NTE), a neural phenyl valerate (PV) esterase defined as the activity resistant to the non-neuropathic paraoxon (diethyl p-nitrophenyl phosphate) and sensitive to the neuropathic mipafox (N,N-diisopropylphosphorodiamidofluoridate) and determined an axonal degeneration of the peripheral nerves and spinal cord (6, 7). The NTE assay is fully validated for toxicological relevance to OPIDN and serves as the monitoring procedure in NTE isolation (2). NTE is, however, also inhibited in the species which are less likely to display clinical and neuropathological changes after OP administration (8).

Neurological signs of OPIDN occur 8 to 21 days after dosing and is characterized by weakness, ataxia, and paralysis in the lower limbs accompanied by neuropathological changes in the CNS and PNS (9-11).

Diisopropylfluorophosphate (DFP) is an organophosphorus compound that completely inhibits NTE *in-vitro* (12). Moreover, LD_50 _value of DFP in mouse is 3.7 mg/kg subcutaneously (13). It was reported that ED_50_ of DFP was only 19% of its LD_50_ (14). Additionally, DFP rapidly inhibits AChE, produces seizures and epilepticus as determined by electroencephalography and causes a high rate of mortality if animals are not treated aggressively to eliminate the peripheral symptoms of cholinergic toxicity (15).

Systemic administration of B_1_, B_6_, and B_12_ vitamin compounds (Neurobion^™ ^Merck, containing 100 mg thiamine chloride hydrochloride, 100 mg pyridoxine hydrochloride, and 1 mg cyanocobalamin in 3mL aqueous solution) alleviates pain in human and animal (16, 17). In addition, in cold-induced nerve injury in rat, administration of vitamins B_1_, B_6_, and B_12_ significantly enhances the regenerative processes in the nerve (18). It was reported that pyridoxine (B_6_) and pyridoxal phosphate (PLP) significantly promoted the neuronal survival of various brain regions in high cell density culture (10^5^ cells/ cm^2^) (19). Also, there seems to be a moderate association between low maternal B_12_ status and the risk of fetal neural tube defects (20) and chronic exposure to methylcobalamin -a vitamin B_12_ analog- protects cortical neurons against NMDA receptor-mediated glutamate cytotoxicity (21). According to studies, LD_50 _of thiamine, pyridoxine, and cobalamin in mouse are 200, 966, and 1600 mg/kg i.p., respectively (18, 22).

Some studies have shown that corticosteroid therapy can be effective in the prevention and treatment of neuropathic pain by inhibition of prostaglandin production (23). For instance, in patients with acute optic neuritis, treatment with a three-day course of high-dose intravenous methylprednisolone (followed by a short course of prednisone) reduces the rate of development of multiple sclerosis over a two-year period (24). 

In this study, effects of neurobion, dexamethasone, and co-administration of both in prevention of DFP–induced delayed neuropathy in mice were investigated. 

## Experimental

*Chemicals*

Materials were obtained from companies as follows: diisopropylfluorophosphate (DFP) from Sigma-Aldrich; phenyl valerate from Johnson Matthey Gmbh; Neurobion from Merk; Dexamethasone from Iran Hormone Company; 5, 5′–dithiobis (2-nitrobenzoic acid) from Sigma-Aldrich; thiobarbituric acid from Sigma-Aldrich; trichloroacetic acid from Sigma-Aldrich. Other chemicals and reagents were prepared or obtained in the highest analytical grade.

*Methods*

*Animal studies*

Thirty five adult balb/c male mice (25 to 35 g) were purchased from the center of comparative and experimental medicine of Shiraz University of Medical Sciences (Shiraz, Iran). The mice were housed under conditions of light (12 h) and temperature (25 ± 2 ºC) in animal house, one week before and during experiments. Food and water were available *ad libitum* throughout experiments. All experiments were conducted in accordance with the care and use at laboratory animals under supervision of Ethics Committee of Shiraz University of Medical Sciences. 

The animals were assigned randomly into 5 groups with 7 mice in each group:

Group 1 received no medication (Normal group).

Group 2 received normal saline as i.p. 1 h before DFP 1 mg/kg subcutaneously (Control or DFP group).

Group 3 received neurobion in 150 mg/kg i.p. according to vitamin B_1_ and 1 h later, DFP 1 mg/kg subcutaneously (Neurobion group).

Group 4 received dexamethasone in 2 mg/kg i.p. and 1 h later, DFP 1 mg/kg subcutaneously (Dexamethasone group).

Group 5 received Neurobion in 150 mg/kg i.p. according to vitamin B_1_ and Dexamethasone in 2 mg/kg i.p. and finally 1h later, DFP 1 mg/kg subcutaneously (Neurobion + Dexamethasone group).

*Tissue and blood sample*

Three weeks after injections (DFP) - neurological signs of OPIDN occurred 8 to 21 days after dosing-, the animals were killed by decapitation under deep anesthesia induced by thiopental (40 mg/kg). Blood samples were taken, centrifuged and the sera were stored at -20 °C. Their brains were excised and stored at -70 °C. Sciatic nerves were excised and kept in formaldehyde 10% for histopathological evaluation.

*Assay of NTE and AChE inhibition*

NTE is considered to be that portion of the PV-hydrolyzing activity which is insensitive to paraoxon but sensitive to mipafox and DFP. Inhibition of mouse brain NTE activity was determined by the procedure developed previously (11). Briefly, the mice brains were homogenized at 10% (w/v) in 50 mM Tris-0.2 mM EDTA buffer, pH = 8. After 15 min of incubation in 37 ºC with phenylvalerate, liberated phenol was analyzed by coupling with 4-aminoantipyrine and absorbance was measured at 510 nm.

Inhibition of serum AChE was assayed by Ellman procedure (25). Briefly, serum was added to phosphate buffer 0.1 M (pH 8.0) and this solution was mixed with Dithiobisnitrobenzoic acid (DTNB) 0.01 M. Then, Acetylthiocholine iodide 0.075 M was added to the solution. Changes in absorbance at 412 nm were recorded for at least 3 min. 

*Quantification of serum glutathione level*

Glutathione level was measured as an indicator of serum thiol using 5, 5′–dithiobis (2-nitrobenzoic acid), according to the spectrophotometric method (26). Briefly, 50μl serum was added to 50 μL phosphate buffer (pH 7.4) and this solution was mixed with 200 μL of 10% TCA to precipitate the proteins. The sample was mixed well and centrifuged at 4000 rpm for 10 min. Then, 200 μL of this supernatant was diluted with 1.5 mL of Phosphate-buffered saline (pH 8.0) and then it was mixed with 100 μL of 5, 5′–dithiobis (2-nitrobenzoic acid). After 10 min, the mixture was vortexed and absorbance was measured at 412 nm to detect the glutathione content.

*2.2.5. Quantification of serum Malondialdehyde (MDA)*

Serum Malondialdehyde (MDA) which is produced during lipid peroxidation was measured as thiobarbituric acid reactive substances (TBARS) or lipid peroxides, according to method of Placer and Cushman (27) as described by Todorova (28) with some modifications. Fifty microliter serum was added to 50 μL phosphate buffer (pH 7.4) and this solution was mixed with 200 μL of 10% trichloroacetic acid (TCA), 75 μL 0.5 NHCl, 150 μL thiobarbituric acid and then, this solution was warmed to 100 ℃ for 45 min. After cooling, it was centrifuged at 5000 rpm for 10 min and absorbance was measured at 532 nm.

*Histopathological evaluation of the sciatic nerve*

Sciatic nerve was fixed in 10% buffer formaldehyde. The fixed sciatic nerves were routinely processed for paraffin embedding. Thin sections (4-5 µm) were cut using a microtome, stained with hematoxylin and eosin (H&E), and examined using a light microscope (29).

*Statistical analyses*

All statistical analyses were performed using GraphPad PRISM software (version 6.01, 2012). Serum AChE, MDA, GSH, and brain NTE data were evaluated by one-way ANOVA; the significance of differences between control and other groups’ mean values at each time point was determined by post hoc pairwise comparisons using the dunnett test (*p* < 0.05).

## Results

**Figure 1:**
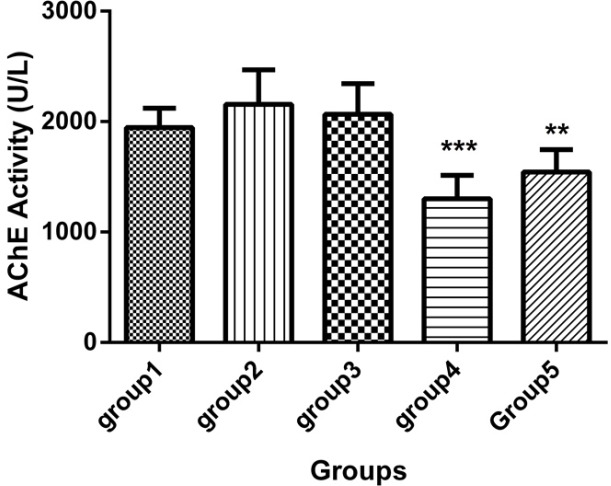
Acetylcholine esterase (AChE) activity in the sera of 5 groups with 7 mice in each (Mean ± SD), 21 days after the last injection. Group1 received no medication. Group 2 received normal saline as i.p. and 1h later, DFP 1 mg/kg subcutaneously. Group 3 received neurobion in 150 mg/kg i.p. and 1h later, DFP 1mg/kg subcutaneously. Group 4 received dexamethasone in 2 mg/kg i.p. and 1h later, DFP 1mg/kg subcutaneously. Group 5 received Neurobion in 150 mg/kg i.p. and Dexamethasone in 2 mg/kg i.p. and 1h later, DFP 1mg/kg subcutaneously. Compared to group 2, AChE activity of group 4 and 5 were significantly differed. **p < 0.01 and ***p < 0.001, one-way ANOVA with post hoc Dunnett's multiple comparisons test.

**Figure 2:**
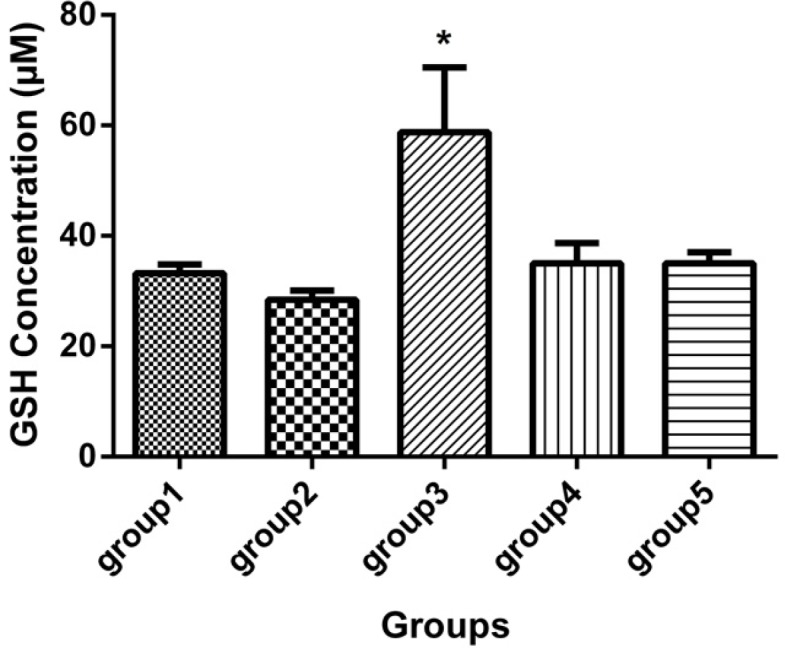
GSH concentration (mcM) in the sera of 5 groups, with 7 mice in each group (Mean ± SD) 21 days after the last injection. Group 1 received no medication. Group 2 received normal saline as i.p. and 1h later, DFP 1 mg/kg subcutaneously. Group 3 received neurobion in 150 mg/kg i.p. and 1h later, DFP 1mg/kg subcutaneously. Group 4 received dexamethasone in 2 mg/kg i.p. and 1 h later, DFP 1mg/kg subcutaneously. Group 5 received Neurobion in 150 mg/kg i.p. and Dexamethasone in 2 mg/kg i.p. and 1h later, DFP 1mg/kg subcutaneously. There was a significant difference between group 3 and group 2. *p < 0.05, one-way ANOVA with post hoc Dunnett's multiple comparisons test.

**Figure 3:**
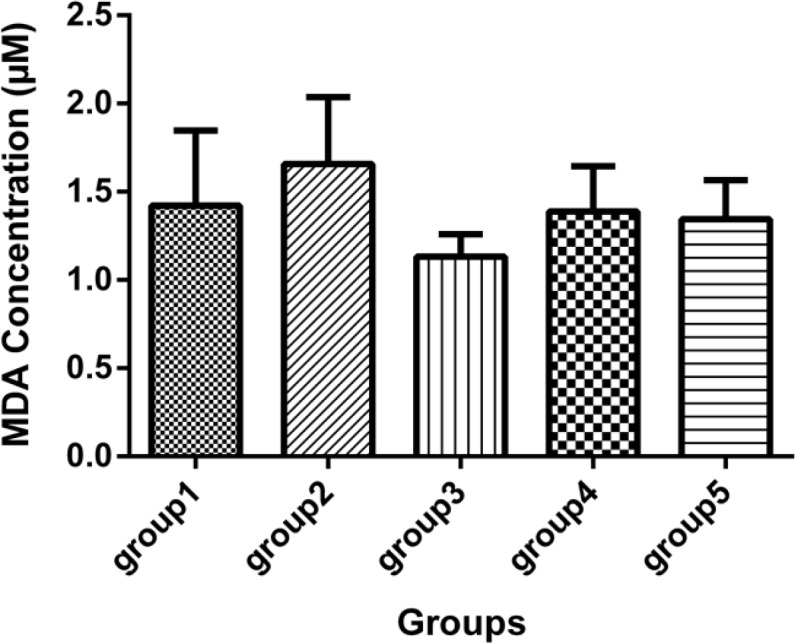
Malondialdehyde concentration (mcM) in the sera of 5 groups, with 7 mice in each group (Mean ± SD) 21 days after the last injection. Group1 received no medication. Group 2 received normal saline as i.p. and 1h later, DFP 1 mg/kg subcutaneously. Group 3 received neurobion in 150 mg/kg i.p. and 1 h later, DFP 1mg/kg subcutaneously. Group 4 received dexamethasone in 2 mg/kg i.p. and 1 h later, DFP 1mg/kg subcutaneously. Group 5 received Neurobion in 150 mg/kg i.p. and Dexamethasone in 2 mg/kg i.p. and 1h later, DFP 1mg/kg subcutaneously. There were no significant differences between the groups compared to group 2 *p* < 0.05, one-way ANOVA with post hoc Dunnett's multiple comparisons test.

**Figure 4:**
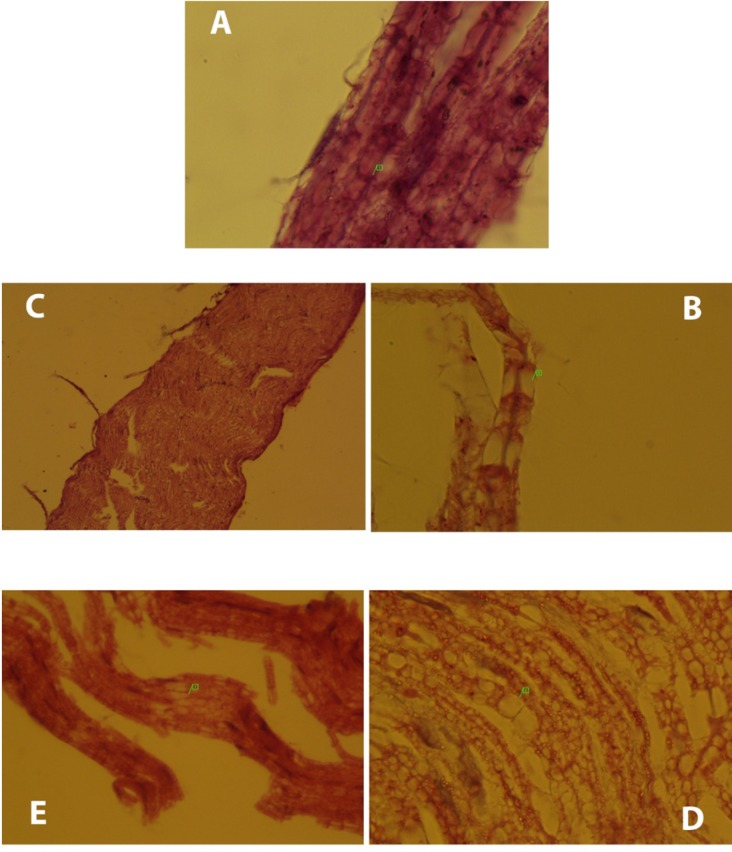
Histopathological changes occurred in the groups compared to the normal group. (A) Normal sciatic nerve had no disruptions and node of Ranvier (1) was clear. (B) Micro cavitation (1) was occurred due to DFP. Moreover, DFP caused myelin loss (group2). (C) Except few disruptions, neurobion prevents degeneration of the nerves due to DFP and the whole normal nerve structure remained intact (group3). (D) DFP caused myelin loss and micro-cavitation (1) and dexamethasone did not affect the harmful effects of DFP (group4). (E) Like group 3, only focal disruption of the nerve fiber (1) was seen due to DFP and neurobion protected the nerve from it (group5). H&E; original magnification, × 1000.

There was one case of mortality in group 2 (DFP) before 21 days. Table 1 and figures 1, 2 and 3 show neuropathy target esterase (NTE) activity as percentage of activity relative to group 2, acetylcholine esterase (AChE) activity, serum glutathione level and serum Malondialdehyde (MDA), respectively. Figure 4 represents histopathological evaluation and staining of the sciatic nerve.

**Table1:** Sensitivity of the mouse brain NTE to *In-vivo *Inhibition, 21 days after the last injection. There were no significant differences between the groups compared to group 2.

**Inhibition relative to DFP group (% ± SD, n = 7)**	
Normal Group	5.0 ± 0.003
Neurobion Group	3.3 ± 0.001
Dexamethasone Group	1.6 ± 0.01
Neurobion + Dexamethasone Group	3.5 ± 0.002

*NTE assay*

Results of NTE assay showed that there was a slight difference, but not significant, between groups 1 and 2, (Table1). Group1 (normal group) had 5% NTE activity more than group 2 (DFP group). Additionally, group 5 (neurobion plus dexamethasone), group3 (neurobion) and group 4 (dexamethason) had only 3.5%, 3.3% and 1.6% NTE activity more than DFP group, respectively (*p *> 0.05). 

*AChE assay*

As shown in Figure 1, acetylcholine esterase (AChE) activity revealed that the dexamethason group and neurobion plus dexamethasone group significantly had lower activity of AChE than the DFP group (*p *< 0.001 and *p *< 0.01, respectively) and the other groups were approximately similar to the DFP group (*p *> 0.05). 

*Serum glutathione level*

According to the GSH assay, it was indicated that the neurobion group had higher concentration of GSH than the DFP group (*p *< 0.05), while the GSH level in other groups did not show any significant difference (Figure 2).

*Serum malondialdehyde*

Malondialdehyde was determined and the results showed that there were no statistically significant difference between the DFP group and other groups (Figure 3).

*H&E staining*

The normal sciatic nerve is shown in Figure 4-A (normal group). Histopathological changes occurred in the DFP group compared to the normal group clearly (Figure 4-B). DFP led to myelin loss and then micro-cavitation in the nerve structure. Sciatic nerve of the neurobion group was obviously in normal structure, despite few nerve disruptions (Figure 4-C). Neurobion was able to prevent the nerve degeneration caused by DFP. In the dexamethason group, demyelination of the nerve was observed (Figure 4-D). Dexamethasone had no inhibitory effect on progress of neuropathy. Also, little interruption was observed in the neurobion plus dexamethasone group (Figure 4-E).

## Discussion

This study describes the effect of vitamins B_1_, B_6_, and B_12_ called neurobion, alone and in combination with dexamethasone on delayed neuropathy caused by diisopropylfluorophosphate. The effect of neurobion and dexamethasone on polyneuropathies was demonstrated in several studies (30, 31). Organophosphate-induced delayed neuropathy (OPIDP) is a rare toxicity resulting from exposure to certain organophosphorus (OP) esters that cause distal degeneration of some axons of both the peripheral and central nervous systems (32, 33). One to four weeks after single or short-term exposures, cramping muscle pain in the lower limbs, distal numbness and paraesthesiae occurred, followed by progressive weakness, depression of deep tendon reflexes in the lower limbs and, in severe cases, in the upper limbs (34-36). In order to determine the OPIDN, assay of NTE level in the brain (9) and histopathological examination of nerve can be helpful (37). Sensitivity of mouse brain NTE to inhibition by DFP *in-vivo* was not different significantly (Table1). In a previous study, 7.8 days was reported as the mean time for return of NTE to 70% normal activity *in-vivo *after exposure to DFP (38). Hence, the results showed that in the DFP group that received only DFP, NTE activity was 5% lower than the normal group as no-medication group (not statistically significant). We did not obtain significant differences among NTE activity after 21 days. This finding is supported by a previous study which was done by Johnson and Henschler (38). Although DFP causes axonal degeneration of the peripheral nerves and spinal cord, neurobion prevents it (39). Neurotoxicity effect of dexamethasone has been reported in different kinds of central and peripheral cells in the nervous system (40). 

Histopathological evaluations of sciatic nerves have clearly shown differences between groups. Although DFP led to demyelination and micro-cavitation in the nerve, neurobion extremely inhibited progress of OPIDP by DFP; hence, the nerve remained intact. It can be said that neurobion acts as a protector against one of the harmful effects of organophosphorus compounds known as OPIDP. Dexamethasone did not affect the harmful effects of DFP and microcavitation observed. As mentioned earlier, dexamethasone has some neurotoxicity effects (40). Neurobion in combination with dexamethasone suppressed the neurodegenerative effect of DFP to some extent. This effect seems to be related to neurobion rather than dexamethasone. We also determined the AChE activity, GSH, and MDA concentration after 21 days. As shown in the AChE activity graph (Figure 1), no difference was seen between DFP and the normal group. It has been reported that DFP has no effect on cholinesterase resynthesis and recovery of cholinesterase activity occuring by 50% after 10 days (41). It seems that AChE activity has returned to normal activity after 21 days.

Although AChE activity in the neurobion group seems to be like the normal group, dexamethasone and dexamethasone in combination with neurobion have diminished the AChE activity compared to DFP (*p *< 0.001 and *p *< 0.01, respectively). It can be related to adverse effects of dexamethasone on acetylcholinesterase activity through nuclear effects (42). Organophosphorus compounds induce oxidative stress and alter antioxidant status in OP poisoning cases (43). There are some antioxidant compounds against free radical damage such as GSH that is the most abundant intracellular thiol-based antioxidant (44). Our finding showed that GSH level in the DFP group was not lower than the normal group (*p *> 0.05); DFP acts as oxidative compound and converts GSH to oxidized form (43), but there was enough time for the cells to compensate for this effect in our study; GSH level in the neurobion group was significantly more than the DFP group, so it can be said that neurobion acts as anti-oxidant compounds that prevent reduction of GSH in the cells. Dexamethasone alone and in combination with neurobion has no significant effect on the GSH level (*p *> 0.05). 

Moreover, the primary targets of reactive oxygen species are cell-membrane polyunsaturated fatty acids, which, in turn, lead to damage in the cell structure and function (45), causing lipid peroxidation, disturbed permeability of membranes and signal transduction (46). Lipid peroxidation can be measured by the MDA level that is the reliable marker end-product of lipid peroxidation in cells. our experiments showed MDA level in the DFP group was slightly higher than the other groups and neurobion, dexamethasone, and their combination prevent lipid peroxidation in cells (statistically not significant). Other studies show that Organophosphorus compounds increase the level of MDA and decrease that of GSH (43). It seems that controversy between our finding and other studies is due to long duration of this study, enough time to repair the damage induced in cell membrane, and also the difference in the animal species used. 

## Conclusion

We suggest that Neurobion^TM^, containing 100 mg thiamine chloride, 100 mg pyridoxine hydrochloride, and 1 mg cyanocobalamin in 3 mL aqueous solution, is better to be added and used in the first aid equipment and techniques for exposure to organophosphorus compounds such as pesticides and chemical warfare.
